# Combined inhibition of Wee1 and Chk1 gives synergistic DNA damage in S-phase due to distinct regulation of CDK activity and CDC45 loading

**DOI:** 10.18632/oncotarget.14089

**Published:** 2016-12-22

**Authors:** Sissel Hauge, Christian Naucke, Grete Hasvold, Mrinal Joel, Gro Elise Rødland, Petras Juzenas, Trond Stokke, Randi G. Syljuåsen

**Affiliations:** ^1^ Department of Radiation Biology, Institute for Cancer Research, Norwegian Radium Hospital, Oslo University Hospital, Oslo, N-0310, Norway

**Keywords:** checkpoint kinase inhibitors, cancer treatment, replication stress, DNA damage, CDK activity

## Abstract

Recent studies have shown synergistic cytotoxic effects of simultaneous Chk1- and Wee1-inhibition. However, the mechanisms behind this synergy are not known. Here, we present a flow cytometry-based screen for compounds that cause increased DNA damage in S-phase when combined with the Wee1-inhibitor MK1775. Strikingly, the Chk1-inhibitors AZD7762 and LY2603618 were among the top candidate hits of 1664 tested compounds, suggesting that the synergistic cytotoxic effects are due to increased S-phase DNA damage. Combined Wee1- and Chk1-inhibition caused a strong synergy in induction of S-phase DNA damage and reduction of clonogenic survival. To address the underlying mechanisms, we developed a novel assay measuring CDK-dependent phosphorylations in single S-phase cells. Surprisingly, while Wee1-inhibition alone induced less DNA damage compared to Chk1-inhibition, Wee1-inhibition caused a bigger increase in S-phase CDK-activity. However, the loading of replication initiation factor CDC45 was more increased after Chk1- than Wee1-inhibition and further increased by the combined treatment, and thus correlated well with DNA damage. Therefore, when Wee1 alone is inhibited, Chk1 suppresses CDC45 loading and thereby limits the extent of unscheduled replication initiation and subsequent S-phase DNA damage, despite very high CDK-activity. These results can explain why combined treatment with Wee1- and Chk1-inhibitors gives synergistic anti-cancer effects.

## INTRODUCTION

Inhibitors of Wee1 kinase are currently in clinical trials for cancer treatment as single agents and in combination with radiation or chemo-therapy [1]. The antitumor effects have traditionally been attributed to the role of Wee1 in preventing G2 checkpoint abrogation [2]. Wee1 is required for the G2 checkpoint through mediating inhibitory phosphorylation of the Tyr-15 residue on CDK1 [3]. Wee1 inhibition leads to abnormally high CDK1 activity, resulting in G2 checkpoint abrogation followed by mitotic catastrophe [4, 5].

However, in addition to its role in G2 checkpoint regulation, Wee1 also plays a major role in suppressing DNA breakage during S-phase [6–8]. Inhibition of Wee1 leads to high CDK1 and CDK2 activity in S-phase followed by unscheduled replication initiation. This results in shortage of replication factors such as nucleotides and replication factor A (RPA), and subsequent replication stalling and endonuclease-induced DNA breakage [7, 9, 10]. Such S-phase damage has been termed “replication catastrophe” [10] and is most likely the major cause behind single-agent antitumor activity of Wee1 inhibitors [11].

Similar as for Wee1, inhibition of Checkpoint kinase 1 (Chk1) causes both G2 checkpoint abrogation and replication catastrophe [12, 13]. Chk1 is thought to regulate these processes mainly through phosphorylation of the Cdc25A phosphatase [7, 13]. Upon Chk1 inhibition Cdc25A is stabilized, giving increased capacity of Cdc25A to remove the inhibitory phosphorylation on CDK1 and CDK2, thereby causing increased CDK activity [14, 15]. Inhibitors of either Chk1 or Wee1 were thus considered to induce replication catastrophe through a common multistep pathway involving high CDK activity, unscheduled replication, replication stalling and subsequent endonuclease-induced DNA breakage [16].

Interestingly, recent preclinical studies have demonstrated synergistic antitumor effects by simultaneous inhibition of Wee1 and Chk1. Cancer cell growth was synergistically reduced both *in vitro* and *in vivo* by combined Wee1 and Chk1 inhibition, as compared to inhibition of each of these kinases alone [17, 18]. Similar effects have been reported in various cancer cell lines of different origin, including ovarian, melanoma, neuroblastoma, leukemia and lymphoma cells, suggesting that combined Chk1/Wee1 inhibition may be a promising approach for cancer treatment [17–22]. However, the molecular mechanisms behind this synergy are not known.

Unbiased large-scale screening has become a powerful tool in biomedical research. Libraries of compounds or siRNAs are widely available and can be applied in functional screens. Whereas siRNA libraries provide strong genetic screens, the advantages of compounds are the possibilities for assays involving rapid kinetics and the direct clinical relevance of many compounds. A typical screen readout involves detection of antibody-staining by automated microscopy [23]. However, recent advances have made it possible to also use flow cytometry in large-scale screens. By connecting a plate loader to the flow cytometer, samples from 384- or 96- well plates can be automatically analysed, allowing rapid and accurate multiparameter analysis of many thousands of cells from each sample [24].

Here, we describe a novel flow cytometry-based screen for compounds that cause increased DNA damage in S-phase when combined with the Wee1 inhibitor MK1775. The screen revealed the Chk1 inhibitors AZD7762 and LY2603618 among the top candidate hits of 1664 tested compounds. Combined inhibition of Wee1 and Chk1 strongly increased replication catastrophe and reduced clonogenic survival. Moreover, the increased DNA damage in S-phase upon Wee1 and Chk1 inhibition correlated much better with loading of the replication factor CDC45 than with the CDK activity of S-phase cells. Our results suggest that Chk1 limits the induction of DNA damage after Wee1 inhibition by suppressing CDC45 loading. These results provide new knowledge about Chk1 function and can explain why simultaneous inhibition of Wee1 and Chk1 kinases give synergistic antitumor effects.

## RESULTS

### Flow cytometry based screen for compounds that cause increased DNA damage in S-phase after Wee1 inhibition

**Figure 1:**
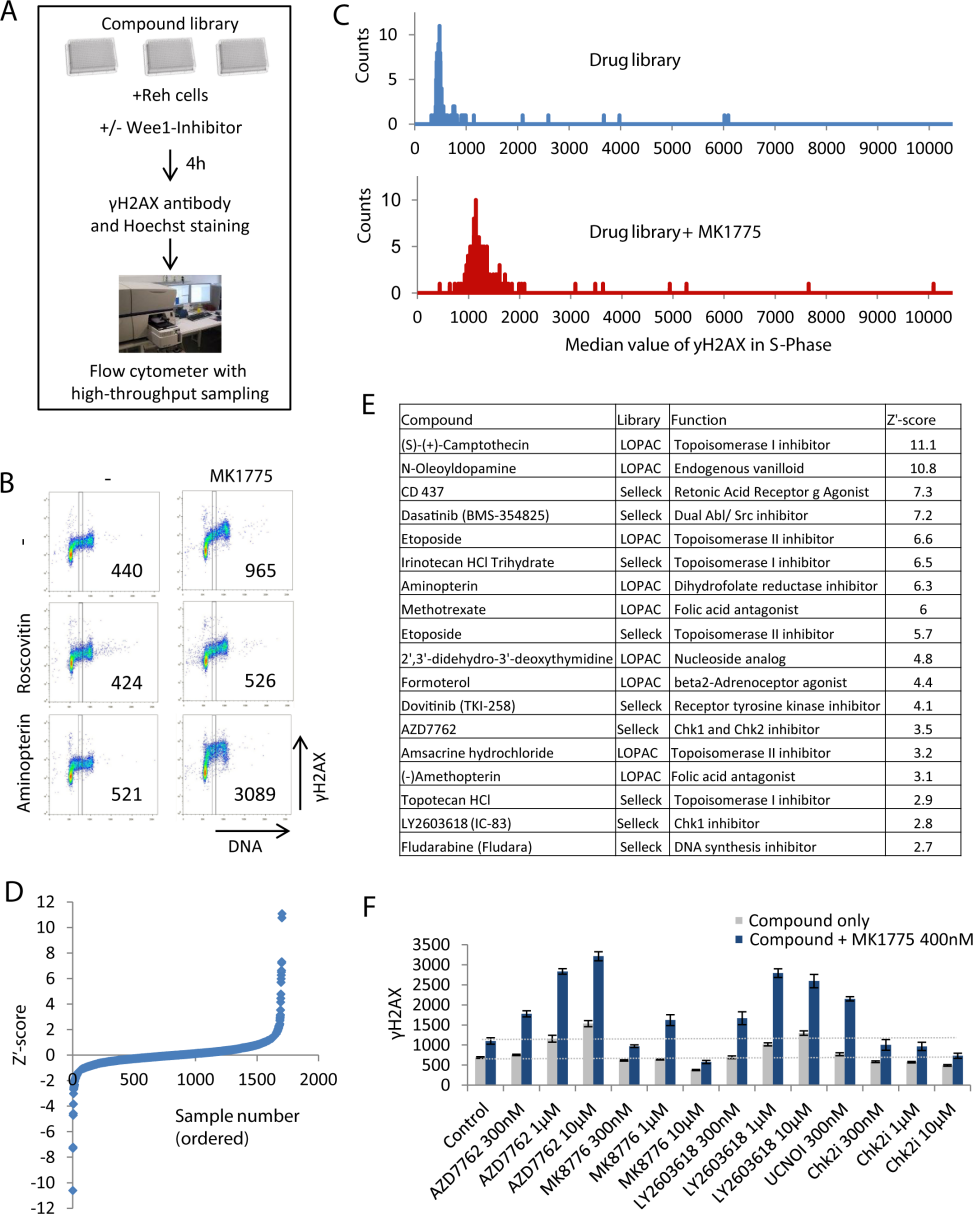
Flow cytometry based screen for compounds that increase DNA damage in S-phase when combined with Wee1 inhibition **(A)** Illustration of screen setup. **(B)** Example of screen results. Density scatter plots are shown for γH2AX versus Hoechst staining (DNA). Vertical lines indicate the region used for quantification of γH2AX levels in S-phase, and numbers indicate median γH2AX levels within this region. **(C)** Example of screen results for a pair of single 384-well plates treated with drug library only (top) and drug library plus 400 nM MK1775 (bottom). The histograms show counts versus γH2AX median values in S-phase. γH2AX median values were obtained as in (B). **(D)** Z’-score values for γH2AXdiff calculated as described in materials and methods. **(E)** List of candidate hits giving synergistic induction of γH2AX in S-phase when combined with MK1775. The compounds with the highest Z’ score in the screen are shown. **(F)** Validation of the results of combined Chk1 and Wee1 inhibition. Reh cells were treated with the indicated concentrations of Wee1 (MK1775), Chk1 (AZD7762, LY2603618, UCN01, MK8776) and Chk2 (PV1019, denoted Chk2i) inhibitors and examined by automated flow cytometry analysis as in A and B. Results are average of three replicates from a representative experiment (three independent experiments gave similar effects). Error bars represent standard deviation. Validation of additional candidate hits is shown in Figure S1D.

To uncover molecular mechanisms behind replication catastrophe and to identify promising drug combinations for cancer treatment, we designed a flow cytometry-based screen combining different compounds with the Wee1 inhibitor MK1775 (Figure 1A). Reh leukemia cells were incubated with the Lopac 1280 or Selleck Cambridge Cancer 384 compound libraries for 4 hours with or without MK1775. DNA damage and cell-cycle profiles were assessed by flow cytometry using an antibody to the DNA damage marker γH2AX and the DNA-stain Hoechst, respectively. Reh cells were used because they grow in suspension at high density, enabling flow cytometry analysis of samples from single wells of 384-well plates without trypsinization. Furthermore, these cells show relatively normal DNA-damage checkpoint responses [25]. To achieve a wide window for detection of compounds that enhance MK1775-induced S-phase DNA damage, a concentration of MK1775 (400 nM) that gave only a small increase in γH2AX staining by itself was chosen (Figure 1B, top panel).

For quantitative analysis of the screen results, a region containing S-phase cells was defined based on the DNA content (Figure 1B), and the median γH2AX level in this region was obtained. As expected, the CDK inhibitor Roscovitine prevented γH2AX induction (Figure 1B, middle panel). In contrast, for example the dihydrofolate reductase inhibitor Aminopterin caused increased γH2AX particularly when combined with MK1775 (Figure 1B, bottom panel). Histograms of the distribution of γH2AX levels from the samples of individual plates showed a clear overall increase in γH2AX levels in plates treated with MK1775 plus drugs compared to drugs only (Figure 1C). Furthermore, a few outliers with higher γH2AX levels than the bulk of the samples were present, representing potential candidate hits.

Notably, a few compounds induced high levels of γH2AX even in the absence of MK1775 (Figure 1C, top panel). We therefore had to discriminate between synergistic versus additive effects of the compounds in combination with MK1775. We calculated the parameter γH2AX_diff_, representing the γH2AX value after treatment with the compound and MK1775 (Figure 1C, bottom panel) minus the γH2AX value after treatment with the compound alone (Figure 1C, top panel). Next, we calculated the Z´-score (described in materials and methods) for the γH2AX_diff_ values (Figure 1D, Supplementary Figure S1A and Supplementary Table S1). The compounds with higher Z´ score values than 2.5 were listed as candidate hits (Figure 1E). The candidate hits included multiple inhibitors of topoisomerase I and II, three folic acid antagonists, two Chk1 inhibitors and a few other drugs with diverse functions (Figure 1E). Some of these compounds caused increased γH2AX levels also in the absence of MK1775 (Supplementary Figure S1B). We also listed the compounds with lower Z´ score values than -2.0, i.e. the compounds that *decreased* the MK1775-induced DNA damage in S-phase (Supplementary Figure S1C and Supplementary Table S1). Notably, five different CDK inhibitors were among the compounds that caused decreased DNA damage (Supplementary Figure S1C). The screen results were thus highly consistent with our previous reports that the S-phase damage induced upon Wee1 inhibition depends on CDK activity [7, 9].

Strikingly, the Chk1 inhibitors AZD7762 and LY2603618 were among the candidate hits. This indicates that the recently reported synergistic antitumor effects of simultaneous Chk1 and Wee1 inhibition [17–22] may be caused by S-phase DNA damage. To validate this result, we performed additional experiments with AZD7762 and LY2603618 and two other Chk1 inhibitors (MK8776 and UCN01), and one Chk2 inhibitor (PV1019). The latter was included in order to discriminate between effects of Chk1 and Chk2, since AZD7762 can inhibit both kinases [26]. All four Chk1 inhibitors, but not the Chk2 inhibitor, caused increased γH2AX in S-phase when combined with MK1775 (Figure 1F). Of note, the highest concentration of MK8776 (10 μM) appeared to decrease γH2AX (Figure 1F), consistent with a CDK inhibitory function of MK8776 at high concentrations [27]. We conclude that combined inhibition of Chk1 and Wee1 causes increased DNA damage in S-phase in Reh cells.

### Wee1 and Chk1 inhibition synergistically enhances replication catastrophe

**Figure 2:**
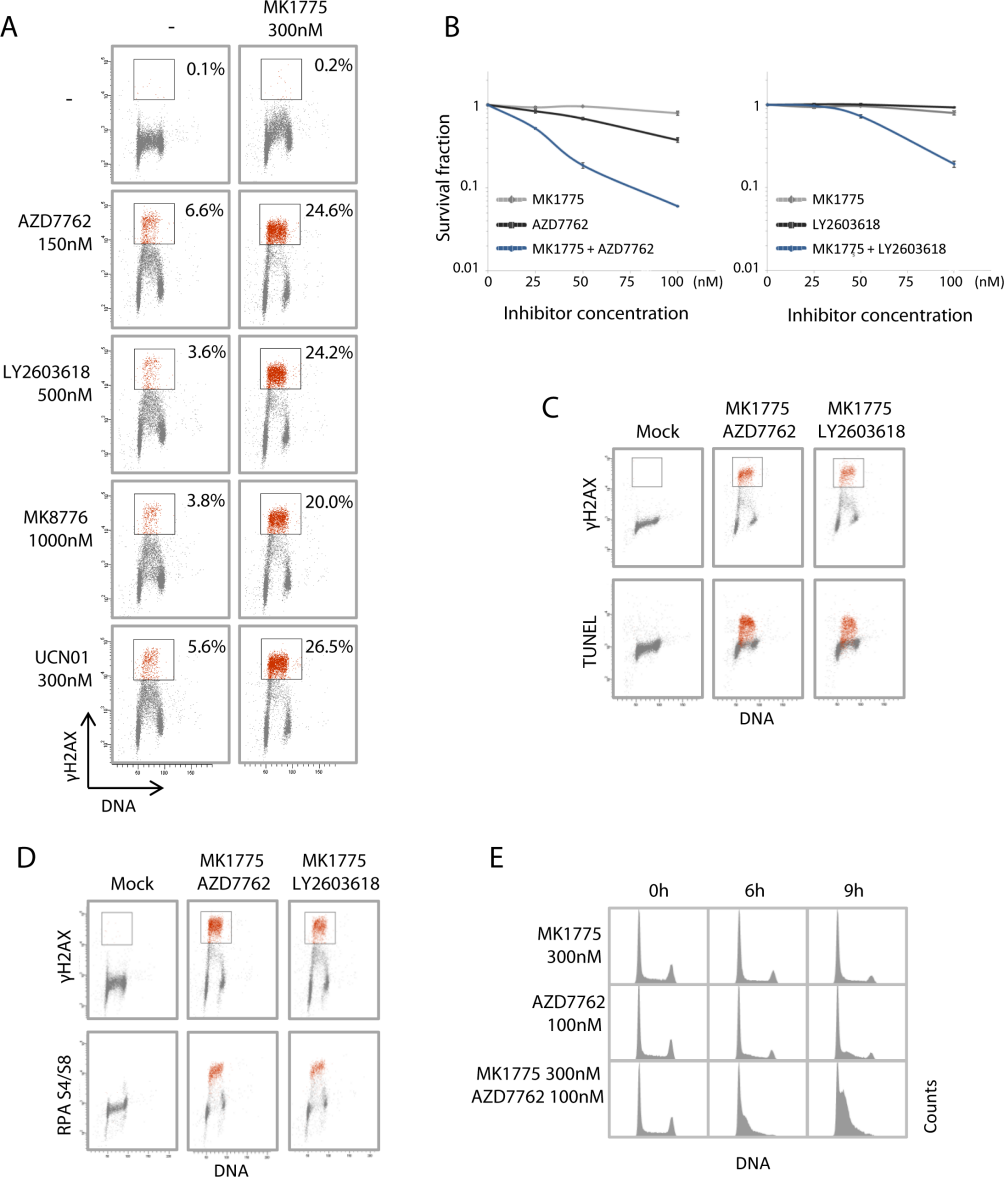
Combined Wee1 and Chk1 inhibition synergistically enhances replication catastrophe **(A)** Flow cytometric analysis of U2OS cells treated for 3 hours with the Wee1 inhibitor MK1775, either of the Chk1 inhibitors AZD7762, LY2603618, MK8776, UCN01, or the combination of MK1775 with each of the Chk1 inhibitors. Scatter plots of γH2AX versus Hoechst (DNA) are shown from a representative experiment. Numbers are the percentage of cells within the indicated region with strong γH2AX signal (red color). **(B)** Clonogenic survival of U2OS cells treated with MK1775 and/or AZD7762 (left) or MK1775 and/or LY2603618 (right), at concentrations 0, 25, 50 or 100 nM for 24 hours. Average survival fractions from three independent experiments are shown. Error bars: SEM (n = 3). **(C)** U2OS cells treated with a combination of MK1775 (300 nM) and AZD7762 (150 nM) or LY2603618 (500 nM) for 3 hours were processed for simultaneous flow cytometric analysis of γH2AX and the TUNEL assay. Scatter plots of γH2AX versus Hoechst (DNA) (top panel) and TUNEL versus Hoechst (bottom panel) are shown. A region defined based on cells with strong γH2AX signals is shown in red color. **(D)** U2OS cells treated as in C processed for simultaneous flow cytometric analysis of γH2AX and phospho-RPA (Ser4/Ser8). A region defined based on cells with strong γH2AX signals is shown in red color. **(E)** U2OS cells treated with MK1775 or AZD7762 or the combination of the two inhibitors as indicated, were stained with Hoechst and analyzed by flow cytometry. DNA histograms are shown.

We next addressed the effects of combined Wee1 and Chk1 inhibition in U2OS osteosarcoma cells. These cells were used in our previous studies of replication catastrophe in response to Chk1 and Wee1 inhibitors as single agents [7, 9, 12]. Treatment of U2OS cells with MK1775 in combination with AZD7762, LY2603618, MK8776 or UCN01 caused a strong increase in induction of γH2AX in S-phase compared to the inhibitors given as single agents (Figure 2A, measured at 3 hours). Clonogenic survival was also strongly reduced (Figure 2B), indicating that the high S-phase DNA damage after the combined treatment resulted in cell death. An exposure time of 24 hours to the inhibitors was chosen for the clonogenic survival assays to resemble a transient delivery of such inhibitors upon *in vivo* single injections. Furthermore, low concentrations of the inhibitors that did not cause much reduction in survival by themselves were used to clearly detect synergistic effects. The cells with strong γH2AX staining were also Tunel-positive (Figure 2C, measured at 3 hours), consistent with severe damage and massive DNA breakage in S-phase. Of note, although the Tunel assay commonly detects apoptosis, similar Tunel-positive S-phase cells after Chk1 inhibition alone did not show other typical features of apoptosis, such as apoptotic nuclear morphology or induction of caspase activity [12]. The S-phase cells with the strongest γH2AX levels also showed high levels of phospho-RPA S4/S8 staining (Figure 2D), indicating presence of single stranded DNA. Moreover, cell cycle analysis at 0–9 hours after treatment revealed massive accumulation of cells in S-phase after combined Chk1 and Wee1 inhibition (Figure 2E), consistent with halted replication. In contrast to the pronounced S-phase effects, the combined treatment gave only a modest increase in the percentages of mitotic cells as examined by the mitotic marker phospho-H3 (Supplementary Figure S2A). Altogether, these data strongly argue that the combined treatment of U2OS cells with Chk1 and Wee1 inhibitors synergistically increases replication catastrophe, and that this may be the major cause for the synergistic anti-tumor effects of these inhibitors.

To address whether similar effects were found in additional cell lines, we examined the lung cancer cell lines H460, A549 and SW900. A previous study showed widely variable growth inhibitory effects of MK1775 alone in these cells, H460 and A549 being the second and third most resistant and SW900 the second most sensitive of a panel of 70 lung cancer cell lines [11]. In response to MK1775 as a single agent, we observed highest induction of γH2AX in S-phase of SW900 and lowest in H460 cells (Supplementary Figure S2B). For these three cell lines, the induction of γH2AX therefore inversely correlates with the published growth inhibition data, consistent with the notion that growth inhibition may be associated with DNA damage in S-phase [11]. All three cell lines showed markedly increased S-phase DNA damage after combined MK1775 and AZD7762 treatment, as judged by either increased levels of γH2AX in S-phase cells or by accumulation of cells with S-phase DNA content (Supplementary Figure S2B). Thus, although the different cell lines tested display variable inherent sensitivity towards Wee1 and Chk1 inhibitors as single agents, the combined treatment consistently strongly enhanced the S-phase DNA damage.

### Measurement of S-phase CDK activity upon Wee1 and/or Chk1 inhibition

**Figure 3:**
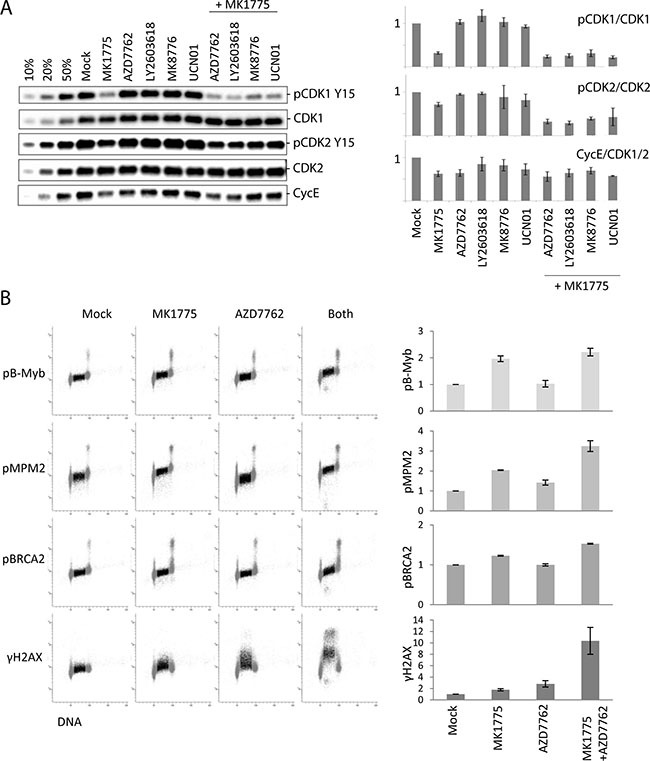
S-phase CDK activity poorly correlates with the extent of DNA damage after Chk1/Wee1 inhibition **(A)** Left: Immunoblot analysis on parallel samples within the same experiment as in Figure 2A collected at one hour after treatment. U2OS cells were exposed to MK1775 (300 nM) and/or AZD7762 (150 nM), LY2603618 (500 nM), MK8776 (1000 nM) and UCN01 (300 nM) for 1 hour. 10%, 20% and 50% of the non-treated sample (Mock) were loaded in the three first lanes to measure the dynamics for each antibody, respectively. Right: Quantifications of phospho-CDK1 (Tyr15) (relative to CDK1), phospho-CDK2 (Tyr15) (relative to CDK2), and Cyclin E levels (relative to CDK1 or CDK2). Error bars: SEM (n = 2 or 3). **(B)** Flow cytometric analysis of CDK-dependent phosphorylations compared to γH2AX in S-phase cells. U2OS cells were treated with MK1775 (600 nM), AZD7762 (100 nM) or both MK1775 (600 nM) and AZD7762 (100 nM) for 1 hour, or left untreated (Mock). The four samples were bar-coded with Pacific Blue before antibody staining with the indicated antibodies. S-phase cells are indicated in dark color. Graphs show average median values in S-phase (relative to Mock) from three independent experiments. Error bars: SEM (n = 3).

Wee1 and Chk1 are both negative regulators of CDK activity, and increased CDK activity is regarded a common mechanism behind replication catastrophe after individual inhibition of each kinase [16, 28]. We previously found that siRNA mediated partial depletion of either CDK1 or CDK2 reduced the S-phase DNA damage upon Wee1, as well as Chk1, inhibition, suggesting that both CDK1 and CDK2 activities contribute to the effects [7]. To address how the single and combined treatments affect CDK activity, we first examined the inhibitory phosphorylation on Tyr15 in CDK1 and CDK2. Immunoblotting was performed on parallel samples within the same experiment as in Figure 2A collected at one hour after treatment. A pronounced reduction in inhibitory phosphorylation on CDK1 was detected after inhibition of Wee1 and Wee1/Chk1, but not after Chk1 inhibition alone (Figure 3A, top panels). The inhibitory phosphorylation on CDK2 was modestly reduced after Wee1 inhibition, and even less so after Chk1 inhibition, whereas the combined treatment showed a small reduction (Figure 3A, middle panels). We also examined Cyclin E levels (Figure 3A, bottom panels). Since Cyclin E is degraded in S-phase in a manner dependent on CDK2 activity, reduced Cyclin E levels may reflect increased CDK2 activity [29]. We observed a small decrease in Cyclin E levels after Chk1 inhibition (Figure 3A, bottom panels), consistent with a slight increase in CDK2 activity. Wee1 inhibition caused a similar or even stronger reduction in Cyclin E levels, but Cyclin E levels were not more reduced after the combined treatment. Taken together with the measurements of γH2AX from Figure 2A, these results suggest that the induction of DNA damage in S-phase does not strictly correlate with CDK1 or CDK2 activity upon inhibition of Wee1 and Chk1.

To further investigate this finding, we developed a flow cytometry method to accurately measure phosphorylation of CDK targets in individual S-phase cells. In this method cells were stained with a DNA-dye together with antibodies to previously reported CDK2 targets (phospho-B-Myb [30, 31] and phospho-BRCA2 [32]) and CDK1 targets (phospho-MPM2 [33]), to measure cell-cycle distribution and CDK activity, respectively (Figure 3B). Addition of Wee1 or Wee1 plus Chk1 inhibitors clearly increased the S-phase signals with all three antibodies (Figure 3B and Supplementary Figure S3). Furthermore, addition of the CDK1 inhibitor RO-3306, the CDK2 inhibitor CVT-313 or the dual CDK1 and CDK2 inhibitor Roscovitine reduced the signals (Supplementary Figure S3A and S3B), indicating that these phosphorylations depend on both CDK1 and CDK2 activity. Cells were also co-stained with an antibody to γH2AX to simultaneously measure the amount of DNA damage. To eliminate potential errors caused by variation in antibody staining, we employed barcoding with pacific blue of sets of four samples.

This method allowed us to assess whether the induction of DNA damage in S-phase correlated with the increase in CDK activity. To investigate early events after adding the inhibitors, cells were treated for one hour. Consistent with our results obtained by immunoblotting, Wee1 inhibition caused a higher increase in CDK activity compared to Chk1 inhibition (Figure 3B). However, Chk1 inhibition caused higher induction of γH2AX. The combined treatment strongly enhanced γH2AX, but showed no or only slight increase in CDK activity compared to after Wee1 inhibition alone (Figure 3B). Thus, the induction of DNA damage in S-phase upon Wee1 and/or Chk1 inhibition does not show an overall correlation with levels of CDK activity.

### Loading of the replication initiation factor CDC45 after Wee1 inhibition is restrained by Chk1

**Figure 4:**
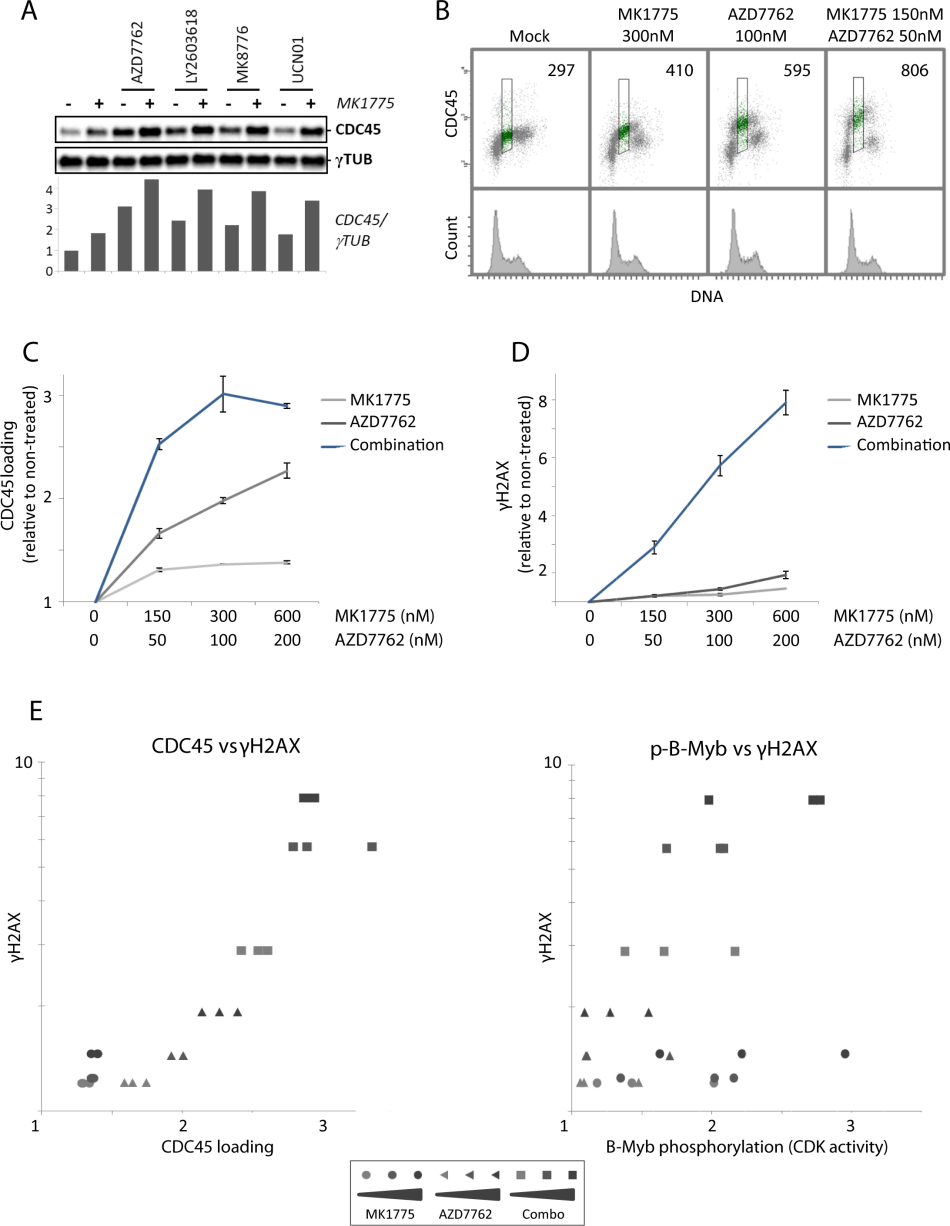
Loading of the replication initiation factor CDC45 after Wee1 inhibition is restrained by Chk1 **(A)** Immunoblot analysis of pre-extracted U2OS cells, treated with MK1775 (150 nM) and/or AZD7762 (50 nM), LY2603618 (250 nM), MK8776 (125 nM) or UCN01 (150 nM) for one hour. Bottom: Quantification of CDC45 levels relative to γ-tubulin (γTUB) levels. Results are from a representative experiment. **(B)** Flow cytometric analysis of U2OS cells treated with MK1775, AZD7762 or the combination of the two inhibitors for 1 hour. Cells were pre-extracted before fixation, bar-coded with pacific blue, and stained with anti-CDC45 antibody and the DNA stain FxCycle Far Red. Numbers indicate median CDC45 signals in S-phase (region shown in green). **(C)** Median CDC45 values in S-phase U2OS cells treated with MK1775 (0, 150, 300, 600 nM) and/or AZD7762 (0, 50, 100, 200 nM) for 1 hour. Pre-extraction, staining and flow cytometric analysis were performed as in B. Error bars: SEM (n = 3). **(D)** Median γH2AX values of S-phase U2OS cells treated with MK1775 and/or AZD7762 for 1 hour similarly as in C. After fixation, the cells were barcoded, stained with anti-γH2AX antibody and the DNA stain FxCycle Far Red, and analyzed by flow cytometry. Error bars: SEM (n = 3). **(E)** Examination of the correlation between CDC45 loading and γH2AX, and CDK activity (as measured by phospho-B-Myb) and γH2AX, for the results shown in C, D and Figure S4C. The values of γH2AX from D were plotted versus the CDC45 values from C (left), or against the p-B-Myb values from Figure S4C (right).

Since unscheduled replication initiation is considered a major cause of replication catastrophe [16, 28], we next examined loading of the replication initiation factor CDC45 after Wee1 and Chk1 inhibition. CDC45 is limiting for replication initiation in humans [34], and we previously showed that partial depletion of CDC45 by siRNA transfection reduced the DNA damage in S-phase upon Chk1 inhibition [12]. Immunoblotting of nonextractable chromatin-bound CDC45 showed a small increase in CDC45 loading after Wee1 inhibition alone, but a markedly higher increase after Chk1 inhibition alone, and the combined treatment further increased CDC45 loading (Figure 4A).

To more accurately measure CDC45 loading in S-phase cells, we conducted flow cytometry analysis with an antibody to CDC45 combined with a DNA-stain after extraction of unbound proteins at one hour after treatment. Again, barcoding of sets of four samples was included to minimize sample-to-sample variations. Wee1 inhibition gave a smaller increase in CDC45 loading compared to Chk1 inhibition, and the combined treatment further increased CDC45 loading (Figure 4B and 4C). Consistent with increased replication initiation, measurements of uptake of the nucleoside analog EdU in S-phase cells followed the same pattern and was also most increased upon the combined treatment (Supplementary Figure S4A and S4B). Levels of γH2AX and phospho-B-Myb were examined in parallel samples within the same experiments to assess induction of DNA damage and CDK activity, respectively. Again, we observed a strong synergistic induction of DNA damage in S-phase upon the combined treatment (Figure 4D), but no synergistic increase of CDK activity (Supplementary Figure S4C). When levels of CDC45 loading in S-phase cells at one hour after treatment with MK1775 and AZD7762 as single agents and in combination were plotted against γH2AX levels, we observed a strong correlation between CDC45 loading and the induction of S-phase DNA damage (Figure 4E, left panel; Pearson coefficient: 0.89 (*p* < 0.0001)). In contrast, γH2AX levels correlated less with CDK activity measurements in the same experiments (Figure 4E, right panel; Pearson coefficient: 0.54 (*p* = 0.005)). These results are consistent with the notion that unscheduled replication initiation is a major cause of the observed S-phase DNA damage in response to Wee1 and Chk1 inhibitors. Furthermore, the increased DNA damage in S-phase upon combined Wee1 and Chk1 inhibition correlates with increased CDC45 loading rather than with higher CDK activity.

## DISCUSSION

**Figure 5:**
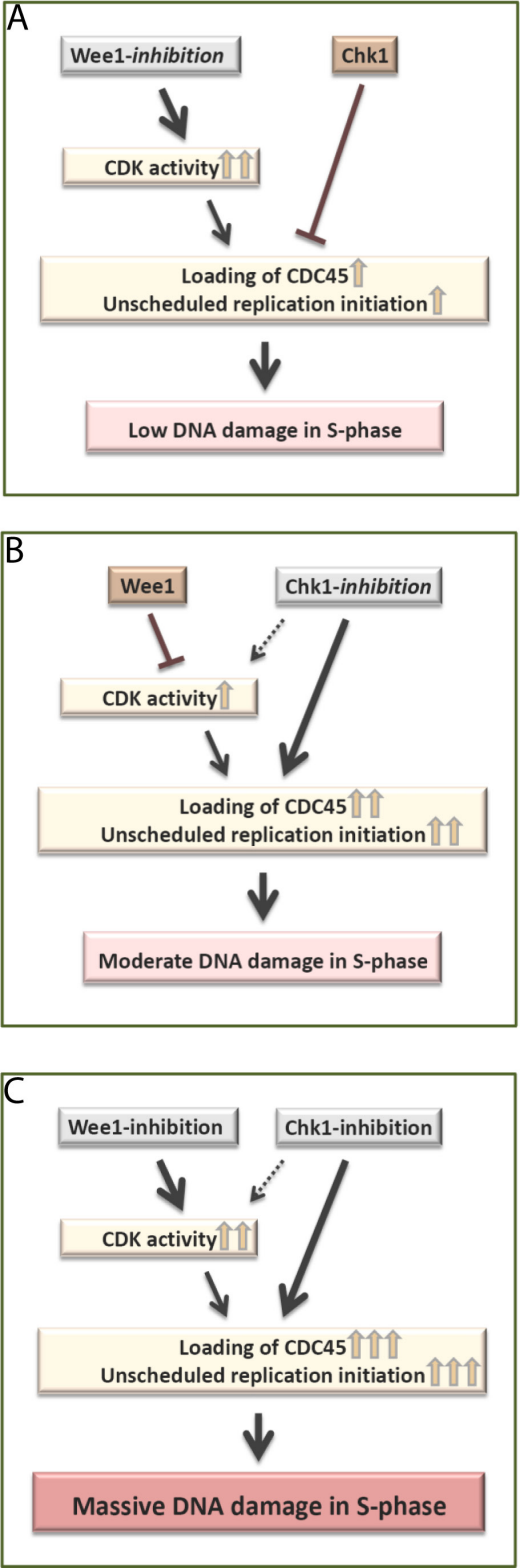
Model **(A)** Wee1-inhibition alone causes very high CDK activity in S-phase, but Chk1 mediated suppression of CDC45 loading limits replication initiation. This results in low levels of S-phase DNA damage. **(B)** Chk1-inhibition alone causes a moderate increase in CDC45 loading, which is limited due to Wee1 mediated suppression of CDK activity. This results in moderate levels of S-phase DNA damage. **(C)** Simultaneous Wee1- and Chk1- inhibition removes the restraints on both CDK activity and CDC45 loading. Consequently, there is a strong increase in CDC45 loading and subsequent massive DNA damage in S-phase.

This study provides an explanation behind the previously reported synergy between Wee1- and Chk1- inhibitors observed in preclinical cancer treatment studies [17–22]. Our results suggest that this synergy is due not only to enhanced CDK activity, but also to an additional, CDK-independent role of Chk1 in regulating CDC45 loading. According to this model, the activity of Chk1 suppresses CDC45 loading and thereby limits replication catastrophe upon Wee1 inhibition alone (Figure 5A). And upon Chk1 inhibition alone, Wee1 suppresses CDK activity and thereby limits CDC45 loading (Figure 5B). However, after inhibition of both kinases, both restraints on CDC45 loading are removed, leading to massive unscheduled replication initiation and subsequent replication catastrophe (Figure 5C).

We have applied a novel assay to measure CDK activity in S-phase (Figure 3B and Supplementary Figure S3). The inclusion of multiple antibodies to previously published CDK2 and CDK1 targets and a DNA-stain to assess cell cycle position, together with the bar-coding approach, enable highly accurate measurements of CDK activity specifically in S-phase cells. This method is an extension of our own previous work with a single CDK target [35], and of others investigating CDK target phosphorylation in mitosis versus interphase [36]. The small increase in S-phase CDK activity upon Chk1 inhibition alone (Figure 3B and Supplementary Figure S4C) is in agreement with previous reports that Chk1 suppresses CDK activity in unperturbed S-phase, through negative regulation of CDC25A phosphatase [12, 15, 28].

Of note, the antibodies used to detect inhibitory phosphorylation (Figure 3A) may not be entirely specific for CDK1 versus CDK2 [29], and some of the CDK-targets in Figure 3B may potentially be phosphorylated by both CDKs. Nevertheless, with all the different readouts we have used to assay CDK activity, our results show that Wee1 is a more potent regulator of S-phase CDK activity compared to Chk1 (Figure 3 and Supplementary Figure S4C). The exact distinction between CDK1 versus CDK2 activity also becomes less important since our previous and present data strongly suggest that both CDK1 and CDK2 contribute to cause the S-phase DNA damage: Depletion of either CDK1 or CDK2 by siRNA reduced the induction of γH2AX in response to Wee1, as well as Chk1, inhibition [7], and inhibitors of either CDK1 (RO-3306) or CDK2 (CVT-313) markedly reduced γH2AX after the combined treatment (Supplementary Figure S3A and S3B, bottom panels).

The mechanisms by which Chk1 can suppress CDC45 loading in the presence of high CDK activity are intriguing. A CDK-independent function of Chk1 in regulation of CDC45 loading was first described in cancer cells exposed to the carcinogen benzo[a]pyrene dihydrodiol epoxide [37]. Recent work has suggested that Chk1 negatively regulates the action of Treslin, a replication factor positively stimulating CDC45 loading [38]. The expression of a mutant version of Treslin that could not bind Chk1 caused increased replication initiation [38]. Based on these findings it seems plausible that Chk1-mediated regulation of Treslin can contribute to suppress CDC45 loading. Notably, Wee1 inhibition causes increased Chk1 activation (Supplementary Figure S4D and [7]), which may further enhance the Chk1-mediated suppression of CDC45 loading.

Interestingly, the combined treatment with Wee1 and Chk1 inhibitors caused massive DNA damage in S-phase without causing premature mitosis (Figure 2 and Supplementary Figure S2A). In contrast, a previous study reported premature mitosis from S-phase upon combined treatment of one breast cancer cell line with MK1775 and AZD7762 [39]. However, the concentration of MK1775 (1 μM) was higher than in our experiments (300 nM) and the incubation time with the inhibitors was longer (8 hours compared to 3 hours in our experiments). Although we cannot exclude that premature mitosis would happen at a later timepoint in U2OS cells, our results clearly show that the massive S-phase DNA damage induced by checkpoint kinase inhibition is not a consequence of premature mitosis.

In this study we present a novel flow cytometry-based screen for compounds that give synergistic DNA damage in S-phase when combined with a Wee1 inhibitor. The strength of our screening approach is the rapid analysis of many thousands cells and accurate measurements of γH2AX levels specifically in S-phase cells. The screen identified several candidate hits, in addition to the Chk1 inhibitors, that have previously been reported to synergize with Wee1 inhibition (Figure 1E). For example, preclinical studies have shown that MK1775 potentiates the cytotoxic effects of Camptothecin [40]. Furthermore, MK1775 combined with Irinotecan Hcl Trihydrate or Topotecan Hcl are currently being tested in clinical trials (Clinical.Trials.Gov). Moreover, three anti-folates were among the candidate hits (Figure 1E and Supplementary Figure S1D). Since anti-folates cause nucleotide deficiency [41], the results of our screen thus support and extend recent work demonstrating a synthetic lethal interaction between Wee1 inhibition and low levels of the ribonucleotide reductase subunit RRM2 [42]. In addition, our results show that MK1775 can enhance the growth inhibitory effects of Dasatinib (Supplementary Figure S1E). Altogether, this strongly suggests that our screen efficiently identifies compounds that synergize with MK1775.

In conclusion, a novel flow cytometry based compound screen revealed synergistic DNA damage in S-phase in cancer cells after simultaneous treatment with Wee1 and Chk1 inhibitors. Our subsequent analysis uncovered that this synergy can be explained by differential functions of Wee1 and Chk1 in regulation of CDK activity and CDC45 loading. Importantly, several of the Wee1 and Chk1 inhibitors used in this study are currently being tested in clinical trials. Our work gives new knowledge about how these inhibitors work as single agents and in combination. These results can help optimizing the future use of Wee1 and Chk1 inhibitors for cancer treatment.

## MATERIALS AND METHODS

### Cell culture and drug treatments

Human NCI-H460 and A549 lung cancer (ATCC) and U2OS osteosarcoma cells were cultured in DMEM (Dulbecco's modified Eagle’s) medium, and SW900 lung cancer and Reh pre-B cell leukemia cells in RPMI (Roswell Park Memorial Institute) medium (both media from Life Technologies), at 37°C in a humidified atmosphere with 5% CO_2_. The media were supplemented with 10% fetal bovine serum (origin South America, Life Technologies) and 1% Penicillin/Streptomycin (Life Technologies). All cell lines (except Reh) were verified by STR (short tandem repeat) technology as described previously [43]. The Wee1 inhibitor MK1775 (AZD1775) was from Merck Calbiochem. The Chk1 inhibitors AZD7762, LY2603618 and MK8776 were from Selleck Chemicals, UCN01 was a gift from R.J. Schultz, National Cancer Institute, and the Chk2 inhibitor PV1019 was from Millipore. The dual CDK1/CDK2 inhibitor Roscovitine and the CDK2 inhibitor CVT-313 were from Cell Signaling, and the CDK1 inhibitor RO-3306 from Merck Calbiochem.

### Flow cytometry-based high-throughput screen

Aliquots of the LOPAC^1280^ Library of Pharmacologically Active Compunds and the Selleck Chem Cambridge Cancer Compound Library distributed in 384-well plates (V-bottom #6008590, Perkin Elmer) were obtained from the Chemical Biology platform, Biotechnology Centre, University of Oslo part of the NOR-OPENSCREEN infrastructure. The screen was conducted at the Flow Cytometry Core Facility, Norwegian Radium Hospital, Oslo University Hospital. A microplate sample processor (Precision XS, BioTek) and microplate washer (EL × 405 Select, BioTek) were used to facilitate the cell seeding, fixation and staining. The final compound concentration was 10 μM. Exponentially growing Reh leukemia cells were seeded at 10^5^ cells/100 μl medium per well. Two parallel plates were processed and analyzed together: one containing compounds only, and the other containing an identical set of compounds plus the Wee1 inhibitor MK1775 (400 nM). Cells were incubated for 4 hours at 37°C/5%CO_2_ and thereafter pelleted by centrifugation. The cell pellets were washed once with PBS, and 70 μl methanol per well was added for fixation. The plates were then stored at −20°C until further analysis. To measure DNA damage in S-phase, cells were stained with an antibody to the DNA damage marker γH2AX together with the DNA stain Hoechst 33258 (Sigma). Cells were incubated for 1 hour at room temperature with 20 μl of mouse anti-phospho-H2AX(Ser139) antibody (05-636, Millipore) diluted 1:1000 in PBS containing 0.2% Tween-20 (Sigma) and 4% milk powder, followed by 30 min incubation with 20 μl of Alexa Fluor 488 anti-mouse IgG diluted 1:1000 (Molecular Probes), and finally resuspended in 80 μl of Hoechst 33258 (1 μg/ml in PBS). The stained plates were stored at 4°C in the dark overnight.

Flow cytometry analysis in the screen was performed with an LSR II flow cytometer (BD Biosciences) equipped with a BD High Throughput Sampler using the FACS Diva Software version 6.1.3 (BD Biosciences) during acquisition. The FlowJo software (FlowJo, LLC) was used during analysis. A region in S-phase was defined based on the Hoechst signal, and the median γH2AX signal within this region was obtained for all samples. To evaluate effects of the drug libraries alone (Supplementary Figure S1B), we calculated the Z-score for each sample (the number of standard deviations away from the median γH2AX sample value of the plate). The calculation of Z-score values separately for each plate enabled comparison of results across plates, despite variations between the plates in overall signal intensity. To identify synergistic effects between MK1775 and the drugs, we calculated the parameter γH2AX_diff_, representing the difference in γH2AX levels between paired samples treated with drug library only, and with MK1775 plus drug library [γH2AX _diff_ = γH2AX _MK1775+drug_ - γH2AX _drug_]. Thereafter, we calculated the Z score for each set of paired plates separately (denoted Z´: the number of standard deviations away from the median sample value of γH2AX_diff_). Samples with higher Z´ score values than 2.5 were considered as candidate hits in the screen (Figure 1E).

### Flow cytometry analysis of CDK targets, CDC45 loading and DNA damage

For analysis of protein phosphorylation, cells were fixed with 70% ethanol and stained with antibodies as described previously [44]. The primary antibodies were mouse anti-phospho-H2AX(Ser139) (05-636, Millipore), rabbit anti-phospho-RPA (Ser4/Ser8)(A300-245, Bethyl Laboratories), rabbit anti-phospho-H3(Ser10) (06-570, Millipore), and three antibodies to CDK targets: rabbit anti-phospho-BRCA2(Ser3291) (AB9986, Millipore), rabbit anti-phospho-B-Myb(Thr487) (ab76009, Abcam) and mouse anti-phospho-Ser/Thr-Pro MPM-2 (05-368, Millipore). Secondary antibodies were Alexa Fluor 488 and 647 (Molecular Probes), Dylight 549 (VectorLabs) and Cy3 (Jackson ImmunoResearch) anti-mouse and anti-rabbit IgG. For analysis of CDC45 loading, cells were pre-extracted and fixed as described in [45], and stained with anti-CDC45 (sc-55569, Santa Cruz) followed by Alexa Fluor 488 anti-mouse IgG. In experiments where median values were measured, barcoding of sets of four samples with pacific blue was used as before [35, 44] to eliminate variation in antibody staining between the individual samples. The DNA stain FxCycle^™^ Far Red (200 nM FxCycle and 0.1 mg/ml RNase A) (Thermo Fisher Scientific) was used in barcoding experiments, and Hoechst 33258 (1.5 μg/ml) in other experiments. The TUNEL TdT kit from Roche, Biotin-16-dUTP (Roche) and Streptavidin-Cy5 (GE Healthcare) were used according to the manufacturer's instruction, combined with antibody staining of anti-phospho-H2AX(Ser139). Flow cytometry analysis was performed on a LSRII flow cytometer (BD Biosciences) using FACS Diva software. The Pearsons correlation coefficient was calculated to quantify the degree of correlation between parameters.

### Clonogenic survival assays

Between 150 and 300 U2OS cells were seeded in 6 cm culture dishes (BD Biosciences) in triplicate with medium containing various concentrations of Wee1 and/or Chk1 inhibitors. After 24 hours, the medium was replaced by 4 ml fresh medium without inhibitors. Cells were then cultured for an additional 13 days, fixed in 70% ethanol and stained with methylene blue. Colonies of 50 or more cells were counted as survivors. Survival fractions were calculated in each experiment as the average cloning efficiency (from 3 parallel dishes) after treatment with the inhibitors, divided by the average cloning efficiency for non-treated cells.

### Immunoblotting

Cells were lysed in SDS boiling buffer (2% SDS, 10 mM Tris-HCl pH 7.5, 100 μM Na_3_VO_4_), and immunoblotting was performed as described previously [43]. The following antibodies were used for blotting: mouse anti-CDK1 (9112, Cell Signaling), rabbit anti-CDK2 (sc-163, Santa Cruz), rabbit anti-phospho-CDK1(Tyr15) (9111, Cell Signaling), rabbit anti-phospho-CDK2(Tyr15) (76147, Abcam), rabbit anti-CyclinE (sc-198, Santa Cruz), mouse anti-CDC45 (sc-55569, Santa Cruz), rabbit anti-phospho-Chk1(Ser296) (2349, Cell Signaling) and mouse anti-γ-Tubulin (T6557, Sigma-Aldrich). In experiments with pre-extraction of unbound proteins, an extraction buffer (0.5% Triton, 20 mM Hepes pH 7.4, 50 mM NaCl, 3 mM MgCl_2_, 300 mM Sucrose) was added for 5 minutes whilst the cells were kept on ice, before one wash in ice-cold PBS, lysis and immunoblotting.

## SUPPLEMENTARY MATERIALS FIGURES AND TABLES




