# Supplementary material for: Combined inhibition of Wee1 and Chk1 gives synergistic DNA damage in S-phase due to distinct regulation of CDK activity and CDC45 loading
Source: Oncotarget. 2016 Dec 22;8(7):10966–79. doi: 10.18632/oncotarget.14089 (PMC5355238; doi:10.18632/oncotarget.14089)
Supplement: Supplementary file 1 [file oncotarget-08-10966-s001.pdf]

Combined inhibition of Wee1 and Chk1 gives synergistic DNA damage in S-phase due to distinct regulation of CDK activity and CDC45 loading

Supplementary Materials

A

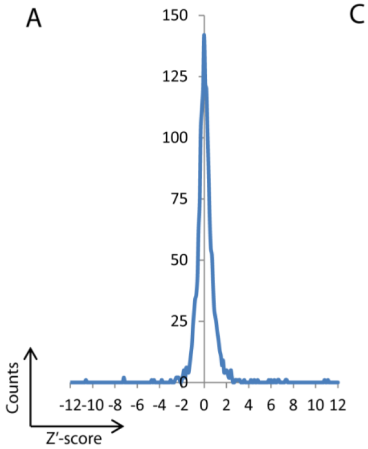

C

Compound	Library	Function	Z'-score
Mitoxantrone	LOPAC	DNA synthesis inhibitor	-10.6
1,10-Phenanthroline monohydrate	LOPAC	Metalloprotease inhibitor	-7.3
Bay 11-7085	LOPAC	Inhibits cytokine induced IkB	-7.2
NSC 95397	LOPAC	Cdc25 phosphatase inhibitor	-4.7
Panobinostat	Selleck	HDAC inhibitor	-4.6
Elesclomol	Selleck	Oxidative stress inducer	-3.8
Purvalanol A	LOPAC	CDK-inhibitor	-3.0
Emetine dihydrochloride hydrate	LOPAC	RNA-Protein translation inhibitor	-2.7
Thapsigargin	LOPAC	intracellular calcium releaser	-2.6
Indirubin-3'-oxime	LOPAC	CDK-inhibitor	-2.5
Kenpaullone	LOPAC	CDK-inhibitor	-2.5
N-Vanillylnonanamide	LOPAC	Synthetic analog of capsaicin	-2.4
Roscovitine	LOPAC	CDK-inhibitor	-2.4
Ro 41-1049 hydrochloride	LOPAC	Monoamine oxidase A inhibitor	-2.3
Cytarabine	Selleck	Antimetabolic agent	-2.3
CGP-74514A hydrochloride	LOPAC	CDK1-inhibitor	-2.1
Wortmannin	LOPAC	Phosphatidylinositol 3-kinase inhibitor	-2.0

B

Compound	Z score (effect of drug in the absence of MK1775)
(S)-(+)-Camptothecin	10.9
N-Oleoyldopamine	10.9
CD 437	9.1
Dasatinib (BMS-354825)	0.5
Etoposide	11.7
Irinotecan HCl Trihydrate	2.7
Aminopterin	0.1
Methotrexate	1.3
Etoposide (VP-16)	5.2
2',3'-didehydro-3'-deoxythymidine	0.5
Formoterol	0.04
Dovitinib (TKI-258)	4.5
AZD7762	1,1
Amsacrine hydrochloride	10.8
(-)Amethopterin	0.03
Topotecan HCl	6.3
LY2603618 (IC-83)	0.5
Fludarabine (Fludara)	3.8

D

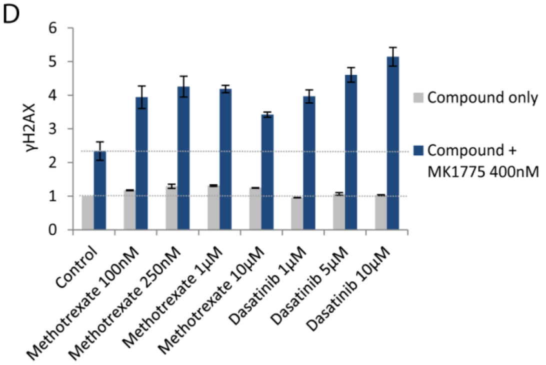

E

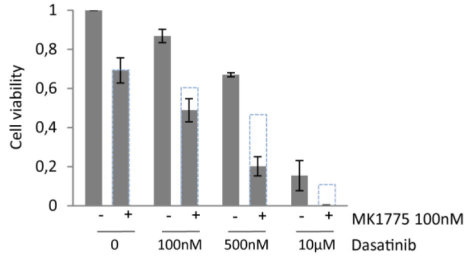

Supplementary Figure S1: (**A**) Plot shows number of samples (counts) versus the Z'-score values from Figure 1D. (**B**) List of Z-scores representing the effects of drug library alone (in the absence of MK1775), for the candidate hits listed in Figure 1E. (**C**) List of candidate hits causing reduced γH2AX in S-phase when combined with MK1775. The compounds with the lowest Z' scores in the screen are shown. (**D**) Validation of the results of combined Dasatinib/MK1775 and Methotrexate/MK1775 treatment. Reh cells were treated with the indicated concentrations of MK1775, Dasatinib and Methotrexate for 4 hours and examined by flow cytometry analysis as in Figure 1B. Error bars: SEM (*n* = 3). (**E**) Viability of Reh cells measured by the CellTiterGlo assay at 72 hours after addition of MK1775 and Dasatinib at the indicated concentrations. Results are average of three independent experiments performed in triplicate wells for each treatment. Blue dashed bars indicate the calculated expected value in the case of additive effects of the two treatments (based on the Bliss independence method). Error bars: SEM (*n* = 3).

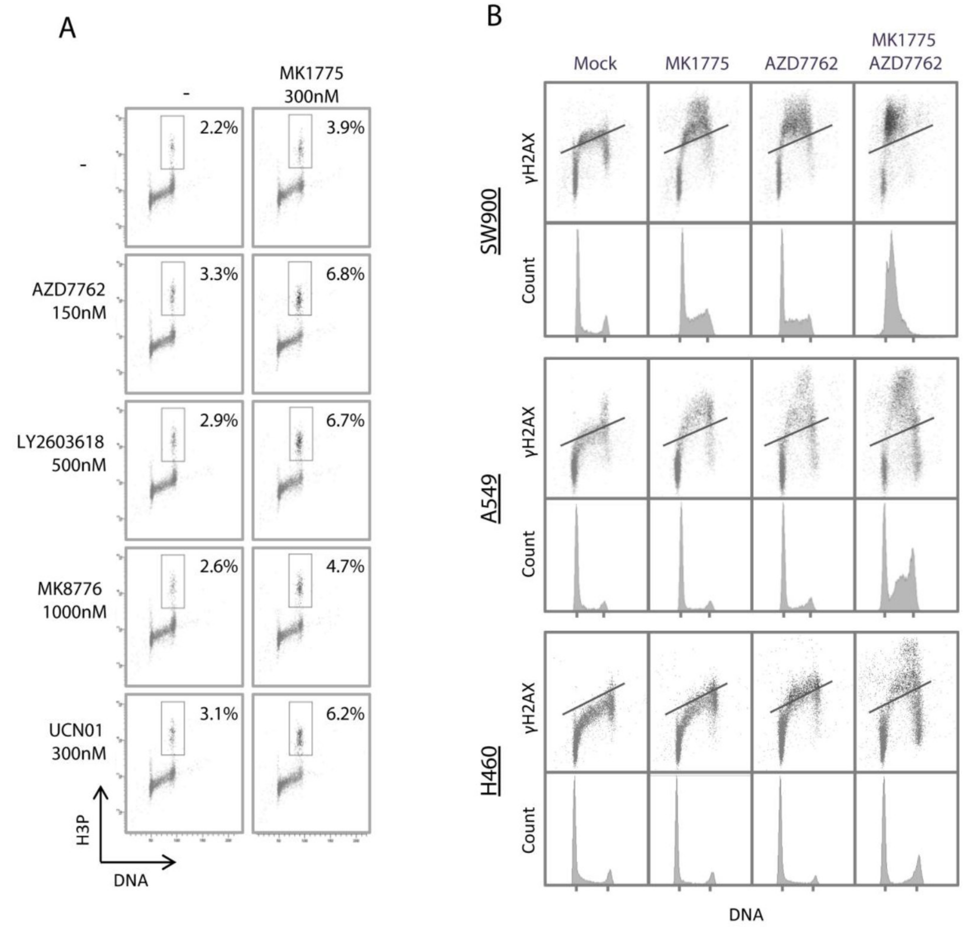

Supplementary Figure S2: (**A**) The samples from Figure 2A were co-stained with phospho-Histone H3 antibody, and analyzed by flow cytometry. Scatter plots of phospho-Histone H3 (H3P) versus Hoechst (DNA) are shown. Numbers indicate the percentage of cells in the region for H3P-positive (mitotic) cells. (**B**) Flow cytometric analysis of SW900, A549 and H460 lung cancer cells treated with MK1775 (300 nM) and/or AZD7762 (300 nM) for 24 hours. Scatter plots of γH2AX versus Hoechst (DNA) and DNA histograms (counts versus DNA) are shown from a representative experiment (three independent experiments were performed with similar results). Lines are included for reference purposes.

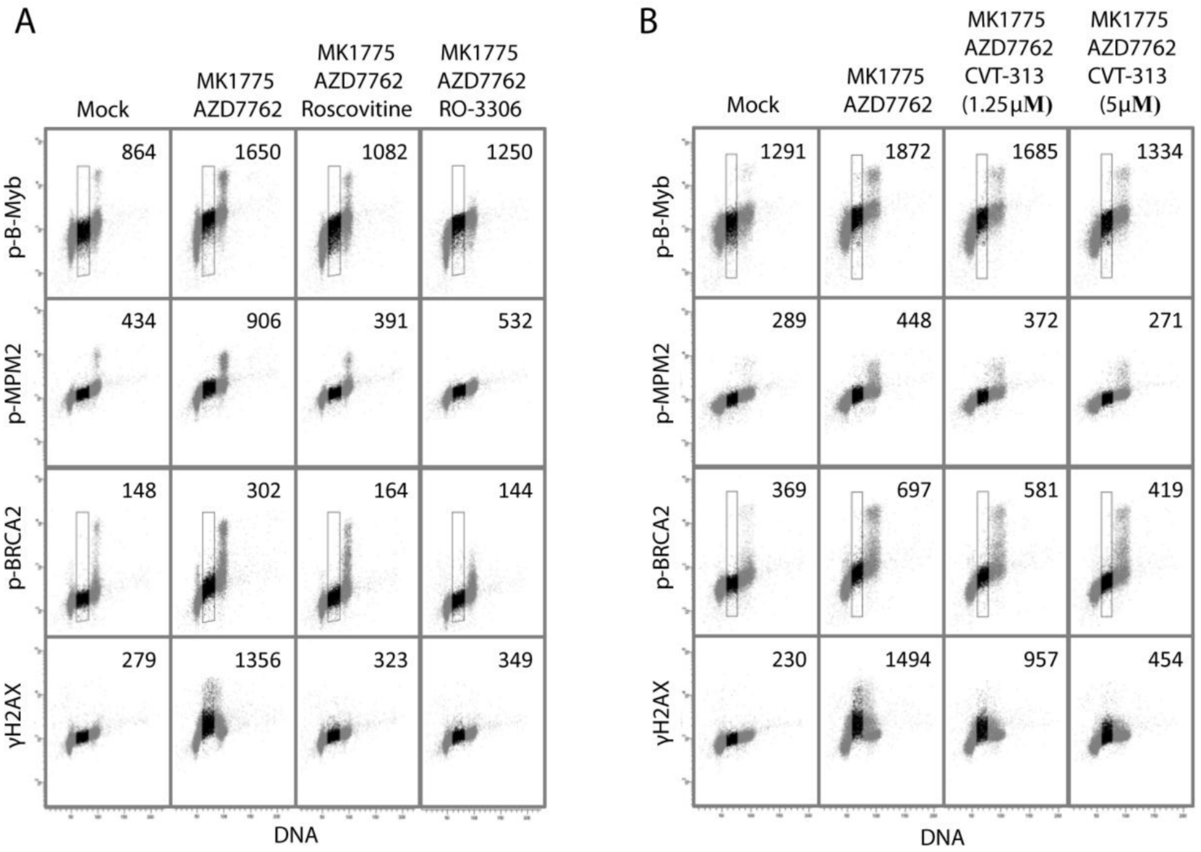

Supplementary Figure S3: (**A**) Flow cytometric analysis of U2OS cells treated for one hour with the combination of MK1775 (600 nM) and AZD7762 (200 nM), alone and in the presence of Roscovitine (25 µM) or RO3306 (10 µM). After barcoding with pacific blue, the samples were split in two and co-stained for phospho-B-Myb and phospho-Ser/Thr-Pro MPM-2 (two top panels), or phospho-BRCA2 and γH2AX (two bottom panels). S-phase regions were defined based on the DNA content (dark color), and numbers indicate median signals in S-phase. (**B**) U2OS cells were treated for one hour with the combination of MK1775 (600 nM) and AZD7762 (200 nM), alone or in the presence of CVT-313 (1.25 or 5 µM). Staining and analysis were performed as in A.

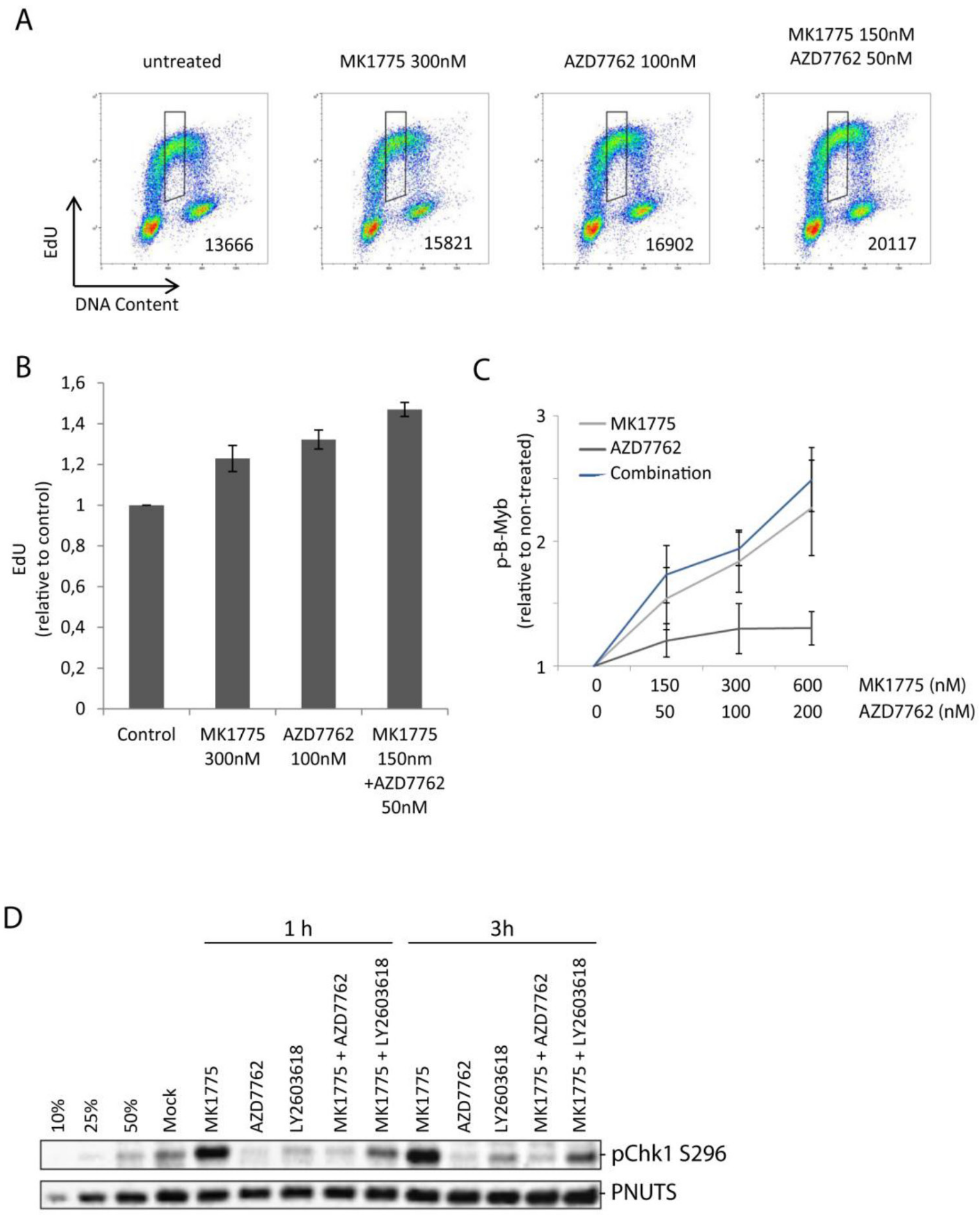

Supplementary Figure S4: (**A**) Flow cytometry analysis of U2OS cells treated with EdU and the indicated concentrations of MK1775 and AZD7762 for 1 hour. Density scatter plots for EdU versus DNA content are shown. Numbers indicate median EdU levels in the mid S phase region minus the average of the median EdU levels of the G1 and G2/M populations. (**B**) Quantification of median EdU levels in mid S phase from 4 independent experiments as in A. Error bars: SEM (n = 4). (**C**) Median phospho-B-Myb values of S-phase U2OS cells treated and analyzed by flow cytometry as in Figure 4D. Error bars: SEM (n = 3). (**D**) Immunoblotting of samples collected at 1 and 3 hours after treatment with MK1775 (300 nM), AZD7762 (150 nM), LY2603618 (500 nM) and combinations as indicated. An antibody to phospho-Chk1 (Ser296) was used to assess Chk1 auto-phosphorylation, and PNUTS (Protein Phosphatase 1 Nuclear Targeting Subunit) levels are included for loading control. 10%, 25% and 50% of the mock sample was loaded in the left three lanes.

CellTiterGlo viability assay

Reh cells were seeded at 5000 cells per well in 96-well plates (white wall/clear bottom plates, Corning) and treated for 72 hours with 10 µm, 500 nM and 100 nM Dasatinib (Sellekchem) in the presence and absence of 100 nM MK1775. Cell viability was assessed by the CellTiter-Glo® Luminescent Cell Viability Assay (Promega) according to the manufacturer's instruction. Luminescence was assessed by a Spark 10M plate reader (Tecan) using the SparkControl Magellan 1.2 software. The Bliss independence method [1] was used to calculate the expected values for additive effects of Dasatinib and MK1775 ($P_{Dasatinib + MK1775} = P_{Dasatinib +} P_{MK177} - P_{Dasatinib} \times P_{MK177}$, where P is the percentage of non-viable cells).

EdU uptake

U2OS cells were treated with MK1775 and/or AZD7762 in the presence of 2 µM EdU for one hour, barcoded with pacific blue (as in Figure 3 and 4) and stained with the Click-iT® EdU Alexa Fluor® 488 Flow Cytometry Assay Kit (ThermoFisher Scientific) and the FxCycleTM Far Red DNA-stain/RNase A. Flow cytometry analysis was performed as in Figures 3 and 4.

REFERENCE

1. Bliss CI. The toxicity of poisons applied jointly. Ann Appl Biol. 1939; 26, 585–615.

Supplementary Table S1: Screen raw data
